# An Improved Electronic Image Motion Compensation (IMC) Method of Aerial Full-Frame-Type Area Array CCD Camera Based on the CCD Multiphase Structure and Hardware Implementation

**DOI:** 10.3390/s18082632

**Published:** 2018-08-11

**Authors:** Hang Ren, Tao Tao Hu, Yu Long Song, Hui Sun, Bo Chao Liu, Ming He Gao

**Affiliations:** 1 Changchun Institute of Optics, Fine Mechanics and Physics, Chinese Academy of Sciences, Changchun 130033, China; renhang10@163.com (H.R.); songyl@ciomp.ac.cn (Y.L.S.); sunh@ciomp.ac.cn (H.S.); liubochao@foxmail.com (B.C.L.); 2 School of Physics, Northeast Normal University, Changchun 130024, China; 3 Chu Kochen Honors College, Zhejiang University, Hangzhou 311058, China; 3170105811@zju.edu.cn

**Keywords:** time delay integration (TDI), image motion, image motion compensation (IMC), charge coupled device (CCD), charge packet transfer, driver timing, non-synchronous effect, drive circuit

## Abstract

In this paper, the performance of the electronic conventional image motion compensation (IMC) method based on the time delay integration (TDI) mode was analyzed using the optical injection formula of charge coupled devices (CCDs). The result shows that the non-synchronous effect of charge packet transfer caused by line-by-line transfer during exposure makes the compensated image dissatisfying. Then an improved electronic IMC method based on the CCD multiphase structure was proposed. In this method, a series of proper driving clocks were applied to drive the charge packet to move electrode-by-electrode during the exposure time, which results in a minimum non-synchronous effect of charge packet transfer. The mismatch of velocity between charge packet transfer and image motion was decreased. The performance of the improved electronic IMC method was also analyzed using the optical injection formula. The modulation degrees of the two methods were compared. The average value of the modulation degree of the improved electronic IMC method was 47/96, greater than the conventional electronic IMC method, which was 1/3. To achieve the improved electronic IMC, the driver timing diagram of the improved electronic IMC method was proposed. This paper presented an improved hardware implementation method for the improved electronic IMC method. Based on the basic FTF4052M drive circuit system, an IMC pulse pattern generator that worked together with the main pulse pattern generator (SAA8103) was added to achieve the improved electronic IMC. Then, the internal structure of the IMC pulse pattern generator was given. A dual pulse pattern generator drive circuit system was proposed. After computer simulation and indoor real shot verification, the compensation effect of the improved electronic IMC method was better than the compensation effect of the conventional electronic IMC method.

## 1. Introduction

### 1.1. Image Motion and Image Motion Compensation (IMC) Methods

When a CCD aerial camera takes an aerial photograph, given the high-speed flight of the aircraft, there is relative motion between the camera and the target during the exposure time, which causes the image of the target on the focal plane to change, that is, image motion. Image motion causes the images of different objects to be aliased to each other, resulting in image degradation due to smearing of images, blurred edges, gray-scale distortion, and reduced contrast and resolution [1]. For example, the reasons for the displacement can be divided into the forward image motion caused by the forward flight of the aircraft; the random image motion caused by changes in the attitude (such as pitch, yaw, and rolling) of the aircraft; aircraft components (such as propellers and engine blocks); camera platforms; and the vibration of the camera itself due to the operation or impact; and the vibration image motion caused by the fluctuation of the air flow. In a vertically-photographing aerial camera, the amount of forward image motion is one order of magnitude higher than that of other image motions, so the main consideration with this type of camera is to compensate for the forward image motion [2]. 

According to the compensation principle and implementation method, the aerial camera’s hardware IMC method can be divided into four categories: the mechanical IMC method [3], the optical IMC method [4], the electronic IMC method [5], and the image-mode IMC method [6]. Each method has its own characteristics.

Mechanical and optical IMC methods require the use of complex and sophisticated optical and mechanical structures [7]; therefore, the camera’s complexity, volume, and weight increases. Due to the use of moving parts, the camera reliability is also reduced. The image-mode IMC method does not have real-time performance [8]. The advantage of the electronic IMC method lies in the use of the CCD device itself. Only the drive circuit of the CCD needs to be modified; therefore, no complicated optical system is needed, which helps to simplify the camera structure [9,10]. Therefore, in the CCD aerial camera, the electronic IMC method is often used in place of other IMC methods.

### 1.2. Electronic Image Motion Compensation (IMC) Method

The electronic IMC method mainly uses a series of CCD charge packet transfer driving techniques to control the CCD’s exposure. It is mainly divided into the IMC method for TDI CCDs, the IMC method for full-frame array CCDs, the IMC method for frame transfer CCDs. The most typical is the IMC method for TDI CCDs [9,10]. 

**Figure 1:**
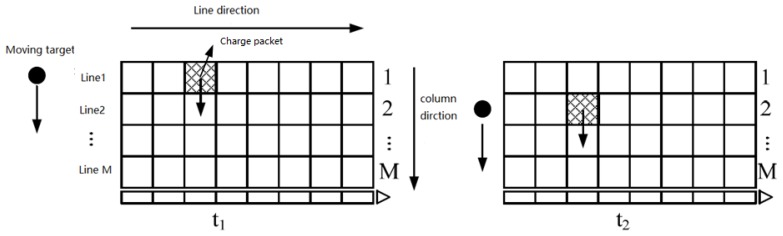
The working principle of the TDI CCD. Black filled circles represent the moving target. The hatched region represents the charge packet generated by the TDI CCD imaging. The number of lines of the TDI CCD represents the number of delay integrals that is M.

The number of lines of the TDI CCD in Figure 1 represents the number of delay integrals that is M, and the target point is imaged and generates a charge packet in one pixel of the first line at time T1. Since the camera and the target have relative motion, the target point at time T2 is vertical. When it moves to the second line, the CCD charge packet is driven to move one line in the direction of the column. As such, the charge packet transferred from the upper line continues to be exposed to the target point. This is repeated until the target point reaches the last line; when the last pixel exposure is finished, the charge packets obtained are the sum of the charge packets of all the TDI pixels exposed to the same target [11,12,13]. In this process, the charge packet moves along the target point, so that the moving target point always generates the charge packet in only one potential well [14,15], and the IMC is realized. 

**Figure 2:**
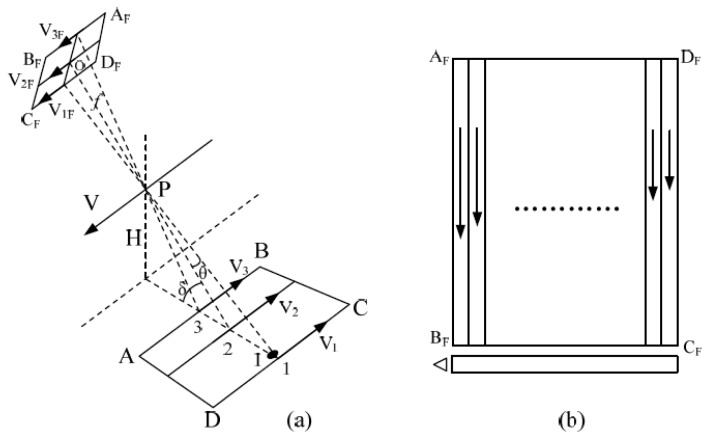
Group IMC schematic of a full-frame CCD. (**a**) CCD IMC during lateral shooting; and (**b**) step IMC.

In 1992, Lareau et al. proposed a full-frame transfer based on the TDI mode in his patent, called the electronic IMC method for full-frame transfer CCDs [3,4]. It is shown in Figure 2. This method divides the full-frame CCD into N groups along the column direction. Each group is driven independently. When the electronic IMC is performed, the charge packets of each group move according to the image motion velocity of the group, thereby improving the compensation accuracy [16,17,18]. 

The principle of the IMC method of the area array CCD is the same as the principle of the electronic IMC method. They are all based on the TDI mode. In the CCD array, the area array CCD can be regarded as an extension of the TDI CCD in the direction of the lines. When IMC is performed, the signal charge packet is synchronously transferred at the velocity of the moving image. The moving image point always produces the charge packet in only one potential well to achieve the purpose of IMC [13,14,15,16].

### 1.3. The Main Contributions of This Article

This paper analyzed the influence of the discreteness of the charge packet transfer on the effect of the conventional electronic IMC method, and proposed an improved electronic IMC method based on the CCD multiphase structure. By modifying the driver timing during the IMC period, the charge packets are transferred between each adjacent electrode. That greatly reduces the non-synchronous effect between the CCD charge packet transfer and the image motion, and it was theoretically proven that the compensation effect was improved. 

To achieve the improved electronic IMC, a vertical transfer driver timing was needed during CCD integration. This paper presented an improved hardware implementation method for the improved electronic IMC method. Based on the basic FTF4052M drive circuit system, we added an “IMC pulse pattern generator” to the system to cooperate the main pulse pattern generator (SAA8103). A dual pulse pattern generator drive circuit system was proposed. The added IMC pulse pattern generator was only used to generate several vertical transfer driver timings required during the exposure period and it is used to forward the driver timings that the SAA8103 generated. This not only supports the electronic IMC function, but also supports the flexible selection of the number of output channels and output modes.

After computer simulation and indoor real shot verification, the compensation effect of the improved electronic IMC method was better than the compensation effect of the conventional electronic IMC method. The improved electronic IMC method was simple and easy to implement, and only needed modification to the vertical transfer driver timing of the CCD during the exposure, but without modifying the circuitry, such as the CCD peripheral driver.

## 2. Problem Formulation

### 2.1. Generation of Non-Synchronous Effect in the Conventional Electronic Image Motion Compensation (IMC) Method

When the TDI CCD performs IMC, the moving velocity of the target image on the image plane must be synchronized with the charge packet transfer velocity. Only in this way can the TDI CCD pixels of each line be exposed to the same target in sequence, and the cumulative charge packet signal of the same target can be finally output [5]. If the moving velocity of the target image on the image plane is not equal to the charge packet transfer velocity, the MTF (modulation transfer function) [17,18] will be degraded. Studies have shown that when the number of the TDI CCD’s stages is *M*, the charge packet transfer velocity $V_{c}$ and the image motion velocity $V_{1}$ should satisfy $\left|V_{c}-V_{1}\right|/V_{c}$ ≤ 2/*M* to ensure that the image quality is not affected [6]. In addition, as the movement of the image is continuous and two-dimensional, the charge packet transfer of the TDI CCD is discrete and one-dimensional. It can only compensate a one-dimensional image of the image. Image motion problems still exist even at full-speed synchronization [19,20].

Regardless of the conventional electronic IMC method of the full-frame CCD or frame-transfer area array CCD, they are all based on the TDI mode. The CCD applies a transfer drive timing to drive the CCD charge packets to move one line. When the charge packets move in sync with the image motion, the image motion can be eliminated. The electronic IMC method requires the charge packet transfer velocity to be synchronized with the image motion velocity, so that one potential well is always exposed to the same image point [21,22]. However, the CCD has a multiphase structure, so it is impossible for the charge packet to be continuously transferred, but the image appears to move continuously. This will inevitably have a non-synchronous effect between the charge packet transfer and the image motion, which will affect the compensation effect. When the conventional electronic IMC method is performed, the charge packets are transferred line-by-line, and the dispersion is large, as analyzed in detail [23,24,25].

### 2.2. Analysis of the Conventional Electronic Image Motion Compensation (IMC) Method

**Figure 3:**
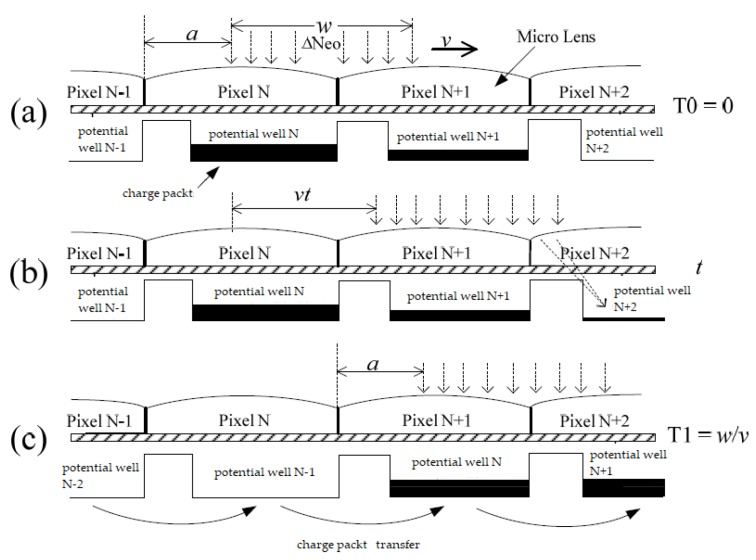
Charge packet transfer diagram of the conventional electronic IMC method based on TDI mode. (**a**) Charge packet transfer diagram at *T*_0_(0); (**b**) charge packet transfer diagram at *t*; and (**c**) charge packet transfer diagram at *T*_1_(*w*/*v*).

Figure 3 is a schematic diagram of the transfer of charge packets under a column pixel of the CCD in the process of the conventional electronic IMC method. In the figure, the pixel shape of the CCD is a square and the width of the side is *w*. Each pixel has a micro lens; its role is to converge the incident light on the surface of the pixel into the potential well to achieve a 100% fill factor. For the sake of simplicity, suppose that a monochromatic light with a photon flow rate of Δ*Neo* and a cell size (a square with a side of *w*) is perpendicularly irradiated on the pixel *N* at time *T*_0_. Without loss of generality, let the edge of the beam be distanced from the edge of the pixel (0 ≤ *a* < *w*), as shown in Figure 3a; this way, the light beam generates charge packets in potential wells *N* and *N* + 1 (if *a* = 0, the charge packet is generated only in potential well *N*). However, due to the image motion, at T1, the beam moved to the right by a pixel-sized distance. At the same time, the CCD was pulsed so that the potential well below the pixel rapidly moved one pixel, thus, the beam still generates charge packets in potential well *N* and potential well *N* + 1. This was repeated until the end of the exposure, as shown in Figure 3c [26].

We can quantitatively analyze the non-synchronous effect of the charge packet dispersion transfer using the optical injection formula of CCD, which is:

(1) $$Q_{IP}=\eta q\Delta N_{eo}AT_{c}$$

where $Q_{IP}$ is the charge quantity of the charge packet that is generated; η is the quantum efficiency of the CCD composition material [27]; *q* is the electronic charge quantity; $\Delta N_{eo}$ is the velocity of the incident photon flow; *A* is the optical area of the photosensitive unit; and $T_{C}$ is the injection time of the photon.

In the process of IMC shown in Figure 3, the charge packet rapidly moved one pixel to the right at *T*_1_ and did not move during the *T*_0_–*T*_1_ time period. During this period, the light beam moved continuously. When *a* > 0, the light beam also generated charge packets in the potential well *N* and potential well *N* + 1, as shown in Figure 3b.

The time from *T*_0_ to *T*_1_ (the beam moves one pixel) was *w*/*v*, and at some time t in this period, the receiving area of the pixel *N* was w(w−a−vt),(0≤t≤(w−a)/v); the light-receiving area of pixel *N* + 1 was w(a+vt)(0≤t≤(w−a)/v) and w(2w−a−vt)((w−a)/v≤t≤w/v), the light-receiving area of the pixel *N* + 2 was w(a−w+vt)((w−a)/v≤t≤w/v). The charge quantity of the charge packet collected in potential well N during this period can be obtained from Equation (4), as follows:

(2) $$Q_{IPN}=\int_{0}^{\frac{w-a}{v}}\eta q\Delta N_{eo}w\cdot (w-a-vt)dt=\frac{1}{2}\eta q\Delta N_{eo}\frac{w}{v}{\left(w-a\right)}^{2}$$

The charge quantity of the charge packet collected in potential well *N* + 1 during this period can be calculated as follows:

(3) $$Q_{IP(N+1)}=\int_{0}^{\frac{w-a}{v}}\eta q\Delta N_{eo}w\cdot (a+vt)dt+\int_{\frac{w-a}{v}}^{\frac{w}{v}}\eta q\Delta N_{eo}w\cdot (2w-a-vt)dt=\frac{1}{2}\eta q\Delta N_{eo}\frac{w}{v}(w^{2}-2a^{2}+2aw)$$

The charge quantity of the charge packet collected in potential well *N* + 2 can be calculated as follows:

(4) $$Q_{IP(N+2)}=\int_{\frac{w-a}{v}}^{\frac{w}{v}}\eta q\Delta N_{eo}w\cdot (a-w+vt)dt=\frac{1}{2}\eta q\Delta N_{eo}\frac{w}{v}a^{2}$$

The above three formulas give the charge quantities of charge packets generated when they are transferred one time, but when the photon injection time $T_{c}$ is longer, the charge packets are constantly transferred. A total of $\frac{T_{c}}{w/v}$ times is transferred in $T_{c}$ time. Moreover, the charge packet transfer velocity is very fast every time [28]. The time spent can be ignored. The charge quantity of the charge packet collected in potential well *N* during $T_{c}$ time can be calculated as follows:

(5) $$Q^{\prime }{}_{IPN}=\frac{T_{c}}{w/v}Q_{IPN}=\frac{1}{2}\eta q\Delta N_{eo}{\left(w-a\right)}^{2}T_{c}$$

The charge quantity of the charge packet collected in potential well *N* + 1 is:

(6) $$Q^{\prime }{}_{IP(N+1)}=\frac{T_{c}}{w/v}Q_{IP(N+1)}=\frac{1}{2}\eta q\Delta N_{eo}(w^{2}-2a^{2}+2aw)T_{c}$$

The charge quantity of the charge packet collected in potential well *N* + 2 is:

(7) $$Q^{\prime }{}_{IP(N+2)}=\frac{T_{c}}{w/v}Q_{IP(N+2)}=\frac{1}{2}\eta q\Delta N_{eo}a^{2}T_{c}$$

if there is a point light beam illuminating the CCD at a distance of every *w* in Figure 3 (spatial frequency is 1/*w*). After the IMC, the charge packet quantities of the adjacent two potential wells are respectively $Q^{\prime }{}_{IP(N+1)}$ and $Q^{\prime }{}_{IPN}+Q^{\prime }{}_{IP(N+2)}$

The degree of modulation of the resulting image is:

(8) $$M=\frac{\left|Q^{\prime }{}_{IP(N+1)}-(Q^{\prime }{}_{IPN}+Q^{\prime }{}_{IP(N+2)}\right|}{Q^{\prime }{}_{IP(N+1)}+Q^{\prime }{}_{IPN}+Q^{\prime }{}_{IP(N+2)}}=\frac{2aw-2a^{2}}{w^{2}}$$

**Figure 4:**
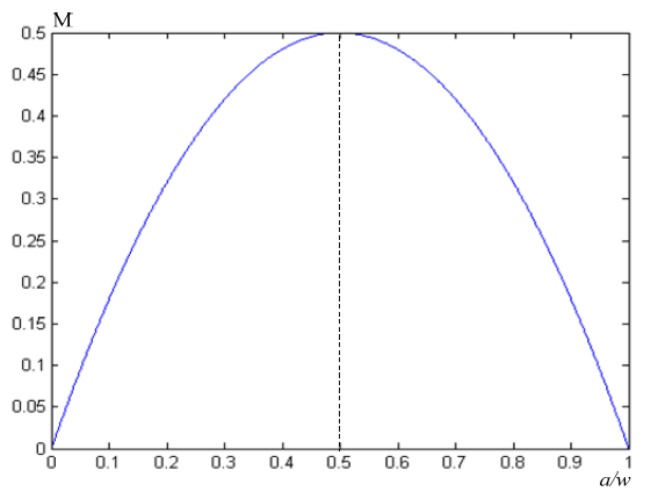
Relationship line between image modulation and image point position based on the conventional electronic IMC method.

The greater the degree of modulation, the easier the image can be resolved, and the lowest value of the modulation that the normal human eye can resolve is generally 0.05. It can be seen from the formula that the image modulation degree obtained by the conventional electronic IMC is related to the starting position of the light beam. As shown in Figure 4, when *a* = *w*/2, M has a maximum value of 0.5, and a single resolution can be more clearly distinguished at this time. When *a* = 0, M has a minimum value of 0. At this point, it is impossible to distinguish a single spot. It can be seen from Figure 4 that, regardless of the beam’s initial position, the modulation degree of the compensated image was less than 0.5, which is unfavorable for human eyes to distinguish, and the compensation effect was not ideal. 

## 3. Improved Electronic Image Motion Compensation (IMC) Method 

### 3.1. Analysis of the Improved Electronic Image Motion Compensation (IMC) Method

The following proposes an improved electronic IMC method based on the CCD multiphase structure. The improved electronic IMC method can reduce the non-synchronous effect, greatly improving the compensation effect [29].

Each pixel of the CCD is composed of multiple electrodes. Take the full frame array CCD FTF4052M as an example. The image area of the FTF4052M uses a four-phase electrode (multiphase) structure. A small pixel is composed of four electrodes, which are P1–P4 in close proximity. We followed certain drive timings. Different levels of P1–P4 are applied so that charge packets in the potential wells can move between P1–P4. Our improved electronic IMC method was to apply high and low levels to P1–P4 according to the specified driver timing so that the charge packets move between each adjacent electrode. This greatly reduces the non-synchronous effect, and enables the charge packet to track the moving image more accurately.

**Figure 5:**
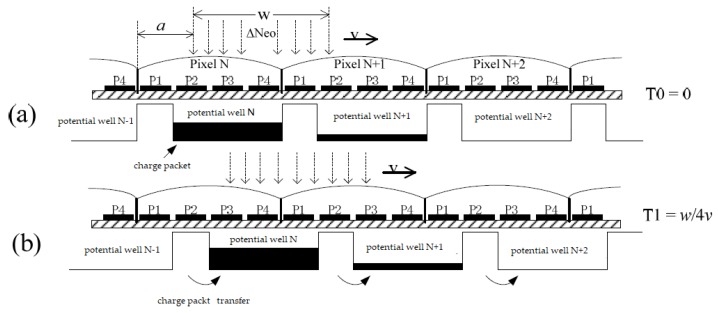
Charge packet transfer diagram of the improved electronic IMC method based on the CCD multiphase structure. (**a**) Diagram of charge packet transfer at *T*_0_; and (**b**) a diagram of charge packet transfer at *T*_1_ (*w*/4*v*).

The improved electronic IMC method principle is shown in Figure 5. We set the CCD to start the exposure at *T*_0_. The single-point beam was irradiated on pixel *N* vertically at time *T*_0_. The edge of the beam was at distance *a* from the edge of the pixel (0 ≤ *a* < *w*), as shown in Figure 5a. As shown, the light beam generated charge packets in the potential well *N* and potential well *N* + 1 (if *a* = 0, only the charge packet is generated in potential well *N*). Due to the IMC (set the image motion rate to *v* and the direction to the right), the beam moves to the right by one electrode width (*w*/4) at *T*_1_ [30]. At this time, we applied a transfer pulse to the CCD to drive the charge packets under the pixel simultaneously. The charge packets in the potential well moved one electrode to the right at the same time; as such, the charge packets follow the moving image, as shown in Figure 5b and, thus, continue to move repeatedly until the end of the exposure [31].

The influence of the discrete transfer of the charge packets in the improved electronic IMC method was analyzed as follows:

(1) When 0 ≤ *a* ≤ 3*w*/4, at a time *t* within *T*_0_−*T*_1_, the receiving area of pixel *N* is w(w−a−vt)(0≤t≤w/4v); the light-receiving area of pixel *N* + 1 is the charge quantity of the charge packet collected in potential well *N* during this time period can be calculated as follows:

(9) $$Q_{IPN}=\int_{0}^{\frac{w}{4v}}\eta q\Delta N_{eo}w(w-a-vt)dt=\frac{1}{32}\eta q\Delta N_{eo}\frac{w^{2}}{v}(7w-8a)$$

The charge quantity of the charge packet collected in potential well *N* + 1 is:

(10) $$Q_{IP(N+1)}=\int_{0}^{\frac{w}{4v}}\eta q\Delta N_{eo}w(a+vt)dt=\frac{1}{32}\eta q\Delta N_{eo}(w+8a)$$

The above two equations give the total charge quantity of the charge packets collected in the potential wells transferred once; however, if the light injection time is extended, the charge packets are transferred $\frac{4T_{c}}{w/v}$ times during $T_{c}$. However, since each charge packet is transferred quickly, the time used is completely negligible. Therefore, the charge quantity of charge packets collected in potential well N during time $T_{c}$ is:

(11) $$Q^{\prime }{}_{IPN}=\frac{4T_{c}}{w/v}Q_{IPN}=\frac{1}{8}\eta q\Delta N_{eo}w(7w-8a)T_{c}$$

The charge quantity of the charge packet collected in potential well *N* + 1 is:

(12) $$Q^{\prime }{}_{IP(N+1)}=\frac{4T_{c}}{w/v}Q_{IP(N+1)}=\frac{1}{8}\eta q\Delta N_{eo}w(w+8a)T_{c}$$

If there is a point light beam illuminating the CCD at a distance of every *w* in Figure 5 (the spatial frequency is 1/*w*) [32], the modulation degree of the image obtained by the improved electronic IMC method is:

(13) $$M=\frac{\left|Q^{\prime }{}_{IP(N+1)}-Q^{\prime }{}_{IPN}\right|}{Q^{\prime }{}_{IP(N+1)}+Q^{\prime }{}_{IPN}}=\frac{\left|3w-8a\right|}{4w}(0\leq a\leq \frac{3w}{4})$$

In the upper form, when *a* = 0, M has a maximum value of 3/4; when *a* = 3*w*/8, M has a minimum value of 0.

(2) When 3*w*/4 < *a* < *w*, the light beam will generate charge packets in potential wells *N*, *N* + 1 and *N* + 2. According to the conventional electronic IMC method, the charge quantities of the charge packets in the three potential wells after the integration time $T_{c}$ are as follows:

(14) $$Q^{\prime }{}_{IPN}=\frac{4T_{c}}{w/v}\int_{0}^{\frac{w-a}{v}}\eta q\Delta N_{eo}w(w-a-vt)dt$$

(15) $$Q^{\prime }{}_{P(N+1)}=\frac{4T_{c}}{w/v}\int_{0}^{\frac{w-a}{v}}\eta q\Delta N_{eo}w\cdot (a+vt)dt+\frac{4T_{c}}{w/v}\int_{0}^{\frac{4a-3w}{4v}}\eta q\Delta N_{eo}w\cdot (w-vt)dt$$

(16) $$Q^{\prime }{}_{IP(N+2)}=\frac{4T_{c}}{w/v}\int_{0}^{\frac{4a-3w}{4v}}\eta q\Delta N_{eo}w\cdot vtdt$$

If there is a point light beam illuminating the CCD at a distance of every *w* in Figure 5 (spatial frequency 1/*w*), the modulation degree of the image obtained by the improved IMC method is:

(17) $$M=\frac{\left|Q^{\prime }{}_{IP(N+1)}-(Q^{\prime }{}_{IPN}+Q^{\prime }{}_{IP(N+2)})\right|}{Q^{\prime }{}_{IP(N+1)}+Q^{\prime }{}_{IPN}+Q^{\prime }{}_{IP(N+2)}}=\frac{\left|56aw-32a^{2}-21w^{2}\right|}{4w}(\frac{3w}{4}<a<1)$$

In the above formula, when *a* = 7*w*/8, M has a maximum value of 0.875.

**Figure 6:**
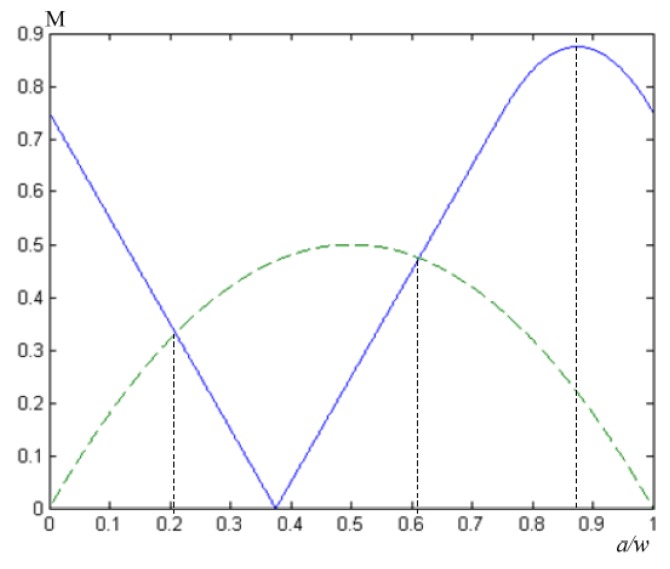
Relationship lines between the image modulation degree and image point position of the conventional electronic IMC method and the improved electronic IMC method. The blue line represents relationship lines between image modulation and image point position of the improved electronic IMC method. The green line represents relationship lines between the image modulation and the image point position of the conventional electronic IMC method.

After the improved electronic IMC, Equations (16) and (20) can be combined to obtain the relationship between the image modulation degree and the starting position of the beam. The curve is shown in Figure 6. To facilitate the comparison [33,34], the modulation degree curve of the conventional electronic IMC method is also plotted. 

From Figure 6, we can see that although at 0.21*w* < *a* < 0.61*w*, the image modulation degree obtained by the conventional electronic IMC method was greater than the image modulation degree obtained by the improved electronic IMC method, the latter was greater than the former in the other regions. The modulation curve of the image obtained by the two IMC methods was integrated separately and divided by the integral length *w*. The average value of the image modulation degree of the conventional electronic IMC method was 1/3, and the average image modulation degree of the improved electronic IMC method was 47/96. The latter was significantly higher than the former, if viewed from the perspective of the human eye alone. In the conventional electronic IMC method, the area of the image modulation degree, which is less than 0.05, accounts for 5.13%; it only accounts for 5% in the improved electronic IMC method. It can be seen that the compensation effect of the improved electronic IMC method was better than the conventional electronic IMC method [35,36].

### 3.2. Driving Time Sequence Analysis

In a vertical photograph CCD aerial camera, the image motion velocity on the CCD focal plane is as follows:

(18) $$v=f\frac{V}{H}$$

where *f* represents the focus of the camera; V is the flying velocity (km/h); and H is the flying height (m).

In the conventional electronic IMC method, we control the charge packet transfer by modifying the vertical transfer drive clock of the area-array CCD photon integration time. When the CCD is normally integrated, each drive clock remains unchanged, and the IMC is performed. The clock is changed at a certain time. The time interval can be obtained by the formula $\Delta t=\frac{w}{v}$ , where w is the pixel size of column direction (mm), and v is the image motion velocity [37].

**Figure 7:**
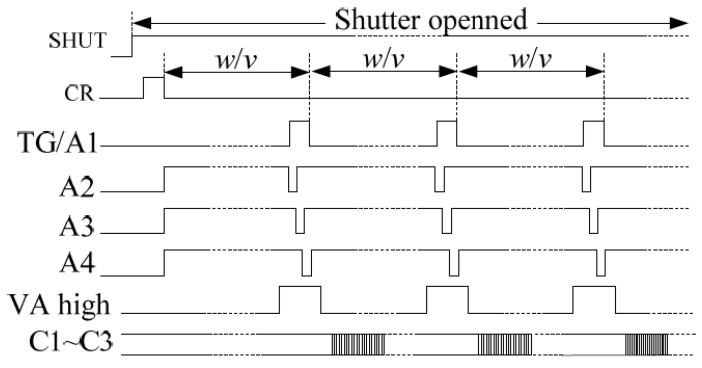
Vertical transfer driver timing during the exposure of conventional electronic IMC. SHUT is the shutter-triggered signal. A1–A4 are the vertical transfer drive clocks, VA high is the high-level transfer clock, and TG is the transfer clock. The CR signal is the charge reset signal of the CCD photosensitive area, and its function is to remove the residual charge of the CCD photosensitive area before the integration begins. C1–C3 are the horizontal transfer drive clocks. w is the pixel size of column direction (mm), and v is image motion velocity (mm/s).

Figure 7 shows the vertical transfer driver timing during the exposure of conventional electronic IMC of the CCDF F4052M. During the exposure, the drive clocks A1–A4 drive the charge packets to move line-by-line and track the moving image. After the transfer is completed, the horizontal clock reads the charge. At the same time, the exposure is over. The work is performed at the normal driver timing [38].

The improved electronic IMC method proposed was also implemented by modifying the vertical transfer drive clock during CCD light integration, but this method shortened the charge packet transfer step width to the CCD pixel electrode width. The drive clock was changed at regular intervals during the exposure, so it can drive the charge packets to move one electrode along the image motion direction. The time interval is calculated by the following equation:

(19) $$\Delta t=\frac{w}{p\cdot v}$$

where w is the pixel size of column direction (mm); v is image motion velocity (mm/s); and p is the number of CCD electrodes of one pixel.

**Figure 8:**
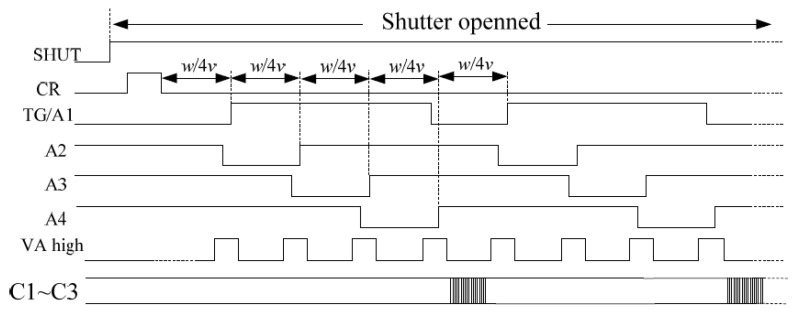
Vertical transfer driver timing during the exposure of the improved IMC. SHUT is the shutter-triggered signal. A1–A4 are the vertical transfer drive clocks, VA high is the high-level transfer clock, and TG is the transfer clock. The CR signal is the charge reset signal of the CCD photosensitive area, and its function is to remove the residual charge of the CCD photosensitive area before the integration begins. C1–C3 are the horizontal transfer drive clocks. w is the pixel size of the column direction (mm), and v is image motion velocity (mm/s).

Figure 8 shows the vertical transfer driver timing during the exposure of the improved IMC during the exposure period, the drive clocks A1–A4 drove the charge packets to move between each adjacent electrode (electrode width is *w*/4) and track the moving image. Every transfer of four electrodes, the horizontal transfer drive clock drove the horizontal register to the output. 

## 4. Hardware Implementation of the Improved Image Motion Compensation (IMC) Method

### 4.1. The Overall Design Scheme of the FTFCCD4052M Drive Circuit System

In order for the FTF4052M to work properly and give full play to its performance, the design of the CCD drive circuit system has become a key issue. As the CCD’s peripheral chips are relatively mature, choosing the DALSA officially recommended driver chipset for the design can greatly reduce the circuit complexity, making the system simple, highly reliable, and excellent in performance [39,40].

**Figure 9:**
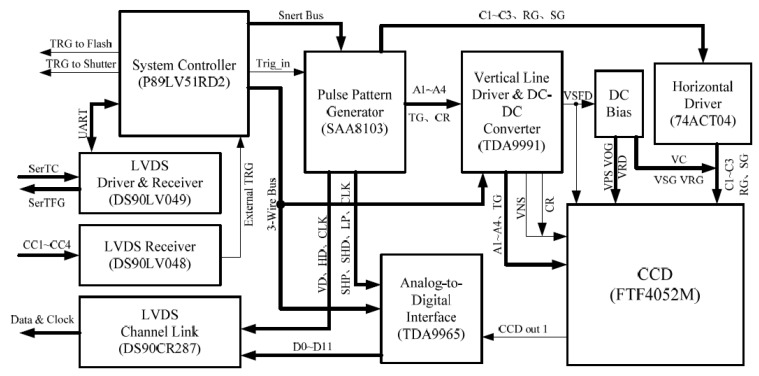
Drive circuit system structure diagram of the FTF4052M. A1–A4 are the vertical transfer drive clocks, VA high is the high-level transfer clock, and TG is the transfer clock. The CR signal is the charge reset signal of the CCD photosensitive area, and its function is to remove the residual charge of the CCD photosensitive area before the integration is started. C1–C3 are the horizontal transfer drive clocks. RG, SG, SHP, SHD, and LP are high-frequency timing pulses closely related to the horizontal pixel shift. CLK is the pixel clock. HD is the line clock signal. VD is the frame clock signal. Trig in is the external trigger signal. RG is the output amplifier reset pulse and SG is the horizontal pixel merge gate drive clock. VNS: Voltage applied to the CCD *n*-type substrate. VPS: Voltage applied to the *p*-type substrate of the CCD.VSFD: DC voltage applied to the CCD output amplifier. VRD: the voltage applied to the drain of the reset transistor of the CCD output amplifier. VOG: Electricity added to the CCD output gate OG.

Figure 9 shows the circuit block diagram of the designed camera driver circuit system. The system consists of a pulse pattern generator (SAA8103 [41,42]), vertical driver (TDA9991), horizontal driver (74ACT04), DC bias circuit, front-end signal processor (TDA9965), system controller (P89LV51RD2), and camera link interface circuit (DS90CR287,DS90LV048/049) [43,44,45,46].

The pulse pattern generator (SAA8103) generates various driver timing pulse signals required by the system. The vertical driver amplifies the vertical transfer driver timing to the drive level signals with sufficient voltage and current drive capability, and generates the main DC bias voltage required by the CCD. The horizontal driver amplifies the horizontal transfer driver timing to a drive level signal that satisfies the CCD operation requirement. The DC bias circuit divides the main DC bias voltage generated by the vertical driver to generate other DC bias voltages required by the CCD. The front-end signal processor performs the correlation acquisition of the analog signal output from the CCD sample, controllable gain amplification, dark level clamp compensation, and analog-to-digital conversion [23]. The camera link interface circuit is responsible for outputting the digital image signals generated by the analog-to-digital conversion from the camera and sending them to the host computer. The system controller is mainly responsible for initializing and configuring the IC in the system during power-up, sending control commands and data, changing the parameters of each IC in real-time, controlling the shooting process, etc. [45,46].

### 4.2. The Hardware Implementation of the Improved Electronic Image Motion Compensation (IMC) Drive Circuit

**Figure 10:**
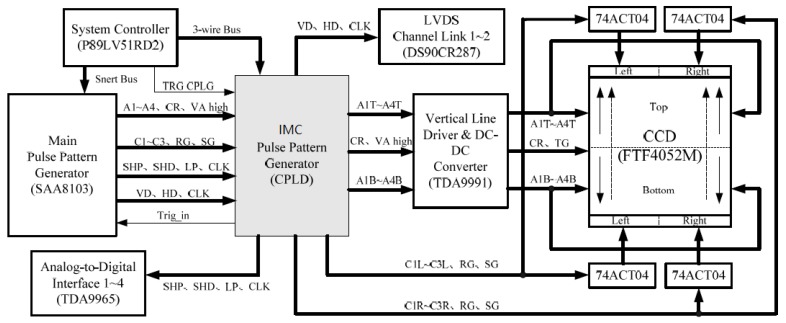
The FTF4052M drive circuit system supporting electronic IMC and multiple outputs. A1–A4 are the vertical transfer drive clocks, VA high is the high-level transfer clock, and TG is the transfer clock. The CR signal is the charge reset signal of the CCD photosensitive area, and its function is to remove the residual charge of the CCD photosensitive area before the integration begins. C1–C3 is the horizontal transfer drive clocks. SHP, SHD, and LP are high-frequency driver timing pulses closely related to the horizontal pixel shift. CLK is the pixel clock. HD is the line clock signal, VD is the frame clock signal. Trig in is the external trigger signal. RG is the output amplifier reset pulse, and SG is the horizontal pixel merge gate drive clock.

To achieve the improved electronic IMC, it is necessary to generate the vertical transfer drive clock (shown in Figure 10) during CCD integration. In the design of the FTF4052M drive circuit system, due to the use of a dedicated driver timing pulse pattern generator (SAA8103) that generates a variety of driver timings, there cannot be any changes to the driver timing of the integration period, so the original drive circuit system needs to be transformed.

The following proposes a double-pulse pattern generator CCD drive circuit system. We added an IMC pulse pattern generator to this basic drive circuit system. It works together with the main pulse pattern generator (SAA8103) in the system. So it can achieve the electronic IMC function. The designed drive circuit system is shown in Figure 10.

In this system, the CCD adopts a four-way parallel output structure. When electronic IMC is performed, all charge packets of the photosensitive area of the CCD are simultaneously moved in one direction (assumed to be upwards, as indicated by the dashed arrow on the CCD in Figure 10). When the charge packet transfer output is performed, the charge packets on the upper half of the photosensitive area move upwards, and the charge packets on the lower half move downwards (as indicated by the solid arrow on the CCD in Figure 10) [43,44,45]; so, the charge packet transfer direction of the lower half changes. To realize the variable-direction charge packet transfer, the CCD is designed to be independently driven in blocks, where the upper half of the photosensitive area is driven by A1T–A4T, the lower half is driven by A1B–A4B, and the left part of the horizontal output register is driven by C1L–C3L. The right part is driven by C1R–C3R, and the charge packet transfer direction of each block is determined by the phase relationship of each drive clock [34].

The IMC pulse pattern generator added to the system is a CPLD. The main function of the device is to generate the vertical transition driver timing required for IMC and it is used to forward and distribute the master driver timings that the main pulse pattern generator (SAA8103) generates. During the CCD exposure, the vertical transfer driver timings (A1–A4 and VA high) required by the FTF4052M are generated by the IMC pulse pattern generator. After being amplified by the TDA9991, the CCD is driven to perform electronic IMC. Other driver timings are generated by the SAA8103, and are forwarded to other parts of the system by the IMC pulse pattern generator. 

### 4.3. Design of the IMC Pulse Pattern Generator

**Figure 11:**
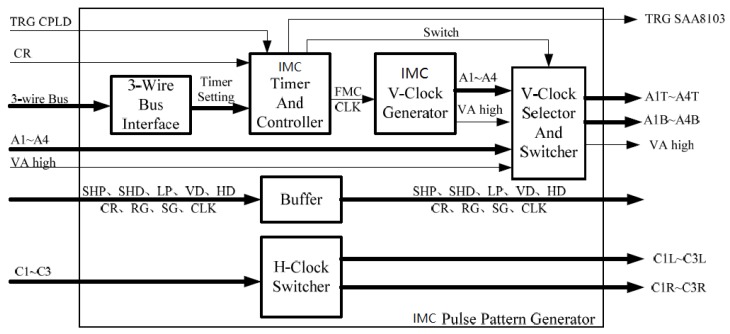
Internal function block diagram of the IMC pulse pattern generator. A1–A4 are the vertical transfer drive clocks, VA high is the high-level transfer clock, and TG is the transfer clock. The CR signal is the charge reset signal of the CCD photosensitive area, and its function is to remove the residual charge of the CCD photosensitive area before the integration begins. C1–C3 is the horizontal transfer drive clock. SHP, SHD, and LP are high-frequency driver timing pulses closely related to horizontal pixel shift. CLK is the pixel clock. HD is the line clock signal, VD is the frame clock signal. Trig in is the external trigger signal. RG is the output amplifier reset pulse and SG is the horizontal pixel merge gate drive clock. TRG is the trigger signal.

The key problem of the transformation is the design of the IMC pulse pattern generator. According to its function, it can be divided into six modules, as shown in Figure 11.

The division and function of each part are as follows:

(1) Three-wire bus interface: Provide a three-wire bus interface to the receive system. Receive the IMC time setting information that the system controller sends.
(2) IMC timer and controller: Generate the timing pulse with interval *w*/4*v* according to the timer setting during exposure, and generate the trigger signal and the timing switch signal of the SAA8103 according to the working status signal;
(3) IMC V-clock generator: Generate the IMC driver timings (A1–A4 and VA high) as shown in Figure 11 based on the timing pulse;
(4) H-clock selector and switcher: Gate the A1–A4 and VA high signals generated by the IMC V-clock generator during exposure. Gate the A1–A4 and VA high signals generated by the SAA8103 during the charge output and idle periods. The gated A1–A4 signals are allocated to A1T–A4T and A1B–A4B, and the phase relationship of each channel is controlled during the allocation process;
(5) H-clock switcher: The horizontal transfer driver timings C1–C3 generated by the SAA8103 are assigned to C1L–C3L and C1R–C3R, and the phase relationship of each channel is controlled during the distribution process.
(6) Signal buffer forwarding module (buffer): Forwards other driver timings generated by the SAA8103.

Using the dual pulse pattern generator structure can make full use of the hardware resources of the original pulse pattern generator of the system, and it can retain the advantages of mature, stable, reliable, and convenient debugging of the original circuit technology. The added IMC pulse pattern generator is used to generate the vertical transfer driver timings required during the exposure and it is used to forward the driver timings generated by the SAA8103. This generator is less difficult to develop, which can greatly reduce the development difficulty, and shorten the development cycle when compared to redesigning the pulse pattern generator. The designed drive circuit system adopts the block-independent driving technology, and the charge packet transfer direction of each block can be set. Therefore, the number of output channels and the output mode can be conveniently selected: When the CCD is applied to low-speed applications, a single output is used to achieve better output consistency. When the CCD is used in high-speed applications, multiple outputs are used to achieve a higher output rate [45,46,47,48].

## 5. Improved CCD Electronic Image Motion Compensation (IMC) Imaging Experiment

### 5.1. The Structure of the Experimental Platform

**Figure 12:**
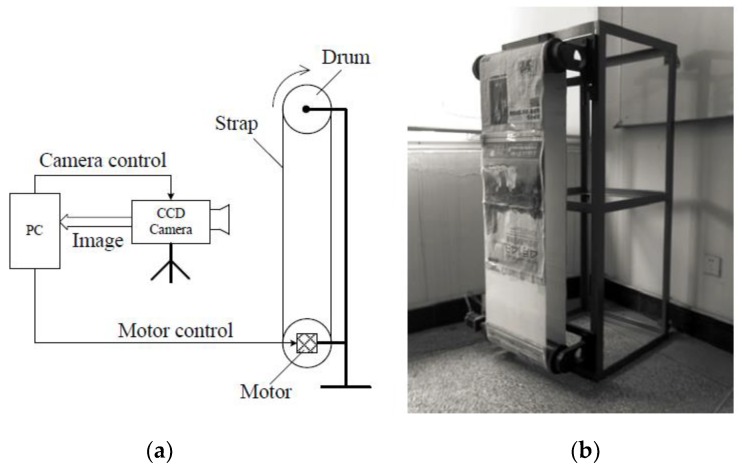
IMC experimental device. (**a**) Block diagram of the experimental device; and (**b**) image motion simulator.

To verify the actual compensation effect of the improved CCD electronic IMC method, an indoor real shot experiment was performed using the designed CCD drive circuit system. The experimental system consisted of a CCD camera, an image motion simulator, and a computer, as shown in Figure 12a. The CCD camera consisted of a designed drive circuit plus a focusable lens; the image motion simulation device consisted of two parallel rollers and a nylon conveyor belt, and the nylon belt was affixed with test pictures. The roller was driven by a DC motor with an adjustable velocity. It was used to simulate the image motion at different velocities, as shown in Figure 12b. A computer was used to control the camera to shoot, change the motor velocity, and send the velocity information to the camera. A Camera Link image capture card was installed in the computer for shooting the image.

### 5.2. Analysis of Two Kinds of Electronic Image Motion Compensation (IMC) in Real-Time

**Figure 13:**
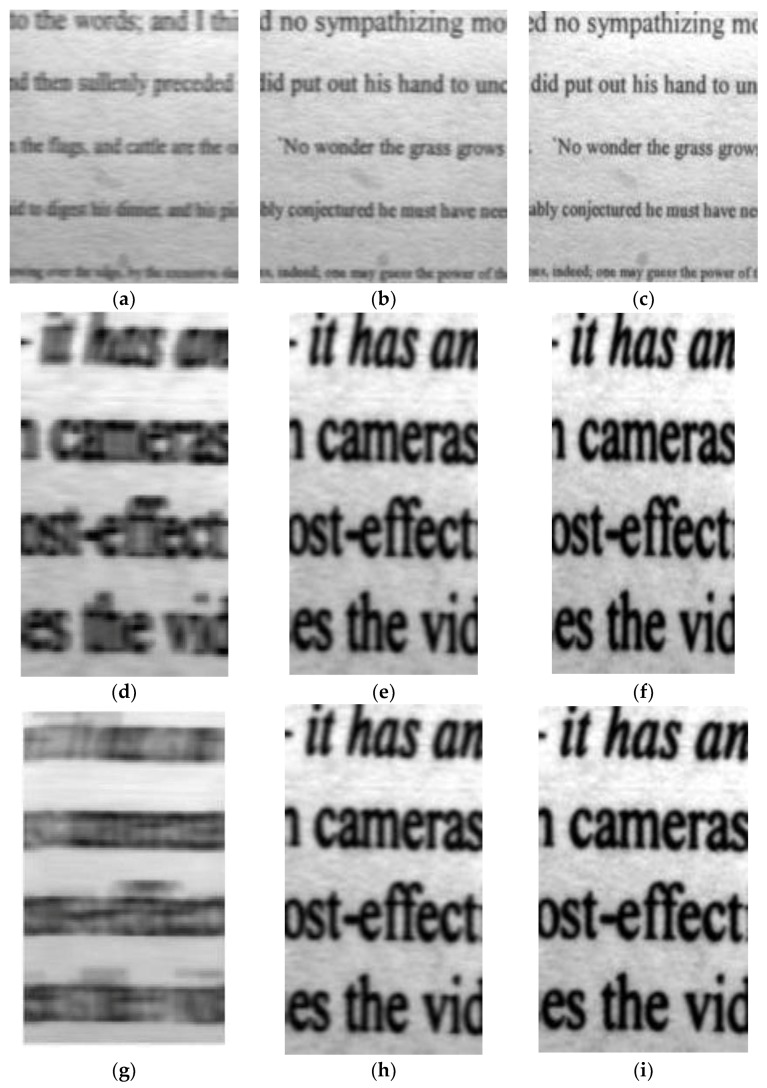
Different renderings of image motion velocity recovery. (a) *v* = 0.00018 m/s without IMC, the image sharpness was calculated as 0.6924; (**b**) *v* = 0.00018 m/s with the conventional electronic IMC method. The image sharpness was calculated as 0.9625; (**c**) *v* = 0.00018 m/s with the improved electronic IMC method. The image sharpness was calculated as 0.9916; (**d**) *v* = 0.0009 m/s without IMC. The image sharpness was calculated as 0.4905; (**e**) *v* = 0.0009 m/s with the conventional electronic IMC method. The image sharpness was calculated as 0.9550; (**f**) *v* = 0.0009 m/s with the improved electronic IMC method. The image sharpness was calculated as 0.990615. (**g**) *v* = 0.0015 m/s without IMC. The image sharpness was calculated as 0.3470; (**h**) *v*= 0.0015 m/s with the conventional improved electronic IMC method. The image sharpness was calculated as 0.9376; and (**i**) *v* = 0.0015 m/s with the improved electronic IMC method. The image sharpness was calculated as 0.9820.

Figure 13 shows the local images of indoor simulation experiments. Figure 13a,d,g are local graphs without IMC; the simulated image motion velocity was 0.00018 m/s, 0.0009 m/s, and 0.0015 m/s; exposure time was 10 ms. Figure 13b,e,h used the conventional electronic IMC method. The compensated images, shown in Figure 13c,f,i are the images compensated by the improved electronic IMC method. To compare the compensation effect of the two IMC methods, we first compared the sharpness of each group of images. In this paper, we used the gray gradient function to evaluate the sharpness:

(20) $$F(n)=\sum_{x}\sum_{y}{\left\{{\left[f(x,y)-f(x+1,y)\right]}^{2}+{\left[f(x,y)-f(x,y+1)\right]}^{2}\right\}}^{\frac{1}{2}}$$

We performed an analysis of the gray gradient function value (Image sharpness) of the image; we brought it into the upper form and normalized it. The results are shown in Table 1.

### 5.3. Discussion

As shown in Table 1, the sharpness of the image in the conventional electronic IMC method was improved by 0.6269, 0.4775, and 0.2765, respectively, and the average value was 0.4603. (0.6269 = 0.9389 − 0.3120, 0.4775 = 0.9576 − 0.4801, 0.2765 = 0.9639 − 0.6874, 0.4603 = (0.6269 + 0.4775 + 0.2765)/3). The image sharpness in the improved electronic IMC method was improved by 0.6747, 0.5144, and 0.3115, respectively. The average value was 0.5002. (0.6747 = 0.9867 − 0.3120, 0.5144 = 0.9945 − 0.4801, 0.3115 = 0.9989 − 0.6874, 0.5502 = (0.6747 + 0.5144 + 0.3115)/3). It is evident that the improved electronic IMC method had a significantly better compensation effect than the conventional electronic IMC method.

## 6. Conclusions

This paper analyzed the limitations of the conventional electronic IMC method based on the TDI mode, and presented the influence of the charge packet transfer discretization on the effect of IMC. A new IMC method based on the CCD multiphase structure was proposed. By modifying the driver timing during the IMC period, the charge packets are transferred between each adjacent electrode. The non-synchronous effect between the charge packet transfer and the image motion was greatly reduced, and it was theoretically proven to improve the compensation effect. The basic drive circuit of the large array CCD FTF4052M was given. Then an IMC pulse pattern generator was added to achieve the electronic IMC function. It worked together with the main pulse pattern generator (SAA8103). Furthermore, a dual pulse pattern generator drive circuit system was proposed. The added IMC pulse pattern generator was only used to generate several vertical transition driver timings required during exposure and it was used to forward the driver timings generated by the SAA8103. This not only supported electronic IMC, but also supported the flexible selection of the output channel number and output mode. Finally, computer simulation and indoor real shot verification were performed, and the experimental results were satisfactory. This method could significantly reduce the non-synchronous effect, thus greatly improving the compensation effect. The mean image modulation of the improved electronic IMC method was 47/96, greater than the conventional electronic IMC method which is 1/3. It was shown that the improved electronic IMC method is better than the conventional electronic IMC method.

## Figures and Tables

**Table 1:** Image sharpness contrast (gray gradient function value).

Image Motion Velocity	No IMC	Conventional Electronic IMC Method	Improved Electronic IMC Method
0.0015 m/s	0.3120	0.9389	0.9867
0.0009 m/s	0.4801	0.9576	0.9945
0.00018 m/s	0.6874	0.9639	0.9989
